# Social Media-Based Health Management Systems and Sustained Health Engagement: TPB Perspective

**DOI:** 10.3390/ijerph16091495

**Published:** 2019-04-27

**Authors:** Dongxiao Gu, Jingjing Guo, Changyong Liang, Wenxing Lu, Shuping Zhao, Bing Liu, Tianyue Long

**Affiliations:** 1 The School of Management, Hefei University of Technology, Hefei 230009, China; 2017170627@mail.hfut.edu.cn (J.G.); cyliang@hfut.edu.cn (C.L.); luwenxing@163.com (W.L.); zhaoshuping1753@126.com (S.Z.); mikehfut0551@163.com (T.L.); 2 The School of Informatics, Computing and Engineering, Bloomington, IN 47405-3907, USA; 3 China Academy of Social Management & School of Sociology, Beijing Normal University, Beijing 100000, China; liubing@bnu.edu.cn

**Keywords:** social media-based health management systems, theory of planned behavior, openness to new experience, sustained health engagement

## Abstract

*Background:* With the popularity of mobile Internet and social networks, an increasing number of social media-based health management systems (SocialHMS) have emerged in recent years. These social media-based systems have been widely used in registration, payment, decision-making, chronic diseases management, health information and medical expenses inquiry, etc., and they greatly facilitate the convenience for people to obtain health services. *Objective:* This study aimed to investigate the factors influencing sustained health engagement of SocialHMS by combining the theory of planned behavior (TPB) with the big-five theory and the trust theory. *Method:* We completed an empirical analysis based on the 494 pieces of data collected from Anhui Medical University first affiliated hospital (AMU) in East China through structural equation modeling and SmartPLS (statistical analysis software). *Results:* Openness to new experience has a significantly positive influence on attitude (path coefficient = 0.671, *t* = 24.0571, *R^2^* = 0.451), perceived behavioral control (path coefficient = 0.752, *t* = 32.2893, *R^2^* = 0.565), and perceived risk (path coefficient = 0.651, *t* = 18.5940, *R^2^* = 0.424), respectively. Attitude, perceived behavioral control, subjective norms, and trust have a significantly positive influence on sustained health engagement (path coefficients = 0.206, 0.305, 0.197, 0.183 respectively, *t* = 3.6684, 4.9158, 4.3414, and 3.3715, respectively). The explained variance of the above factors to the sustained health engagement of SocialHMS is 60.7% (*R^2^* = 0.607). Perceived risk has a significantly negative influence on trust (path coefficient = 0.825, *t* = 46.9598, *R^2^* = 0.681). *Conclusions:* Attitude, perceived behavioral control, subjective norm, and trust are the determinants that affect sustained health engagement. The users’ personality trait of openness to new experience and perceived risk were also found to be important factors for sustained health engagement. For hospital managers, there is the possibility to take appropriate measures based on users’ personality to further enhance the implementation and utilization of SocialHMS. As for system suppliers, they can provide the optimal design for SocialHMS so as to meet users’ needs.

## 1. Introduction

With the rapidly increasing development of mobile Internet and the popularity of social networks, an increasing number of social media-based health management systems (SocialHMS) have emerged in recent years [1]. These social media-based systems have been widely used in registration [2], payment, decision making, chronic diseases management, health information and medical expenses inquiry [3], etc., and they greatly facilitate the convenience for people to obtain health services [4,5]. WeChat is one of the most popular social media platforms in China. In the medical field, the application of WeChat can provide patients with functions such as inquiry, appointment, number taking, payment, etc. As long as they pay attention to the public account of the hospital, they can realize more convenient services in WeChat. For example, in terms of Anhui Provincial Hospital, patients can pay attention to the WeChat public number of the hospital, so that they can not only view the relevant information of doctors and experts in the department but also select an appropriate doctor to make an appointment according to their own symptoms and conditions. What is more, they can make use of other advantages of the public number, such as checking the hospital address and ride information, visiting the waiting team information, checking test results, paying online, and checking medical expenses. By using WeChat as a social media platform to develop a health management system, it is possible for patients to shorten the waiting time, appointment arrangement, registration, and examination in the treatment process, so that they can reasonably arrange the waiting and treatment time. When patients know their waiting time for medical treatment, they can arrange their daily affairs flexibly. With the continuous improvement of social media, the application development of social media-based health management system is constantly changing. In addition, the functions of such systems are getting closer and closer to becoming perfect, and the process is more convenient, and meanwhile, the service is more and more optimized. First, a social media-based health management system can realize the connection between patient information and hospital system data, so that data analysis can provide better personalized medical services for patients. For example, the patient can pay attention to the WeChat public number of the hospital and seek medical treatment through the public number before they come to the hospital. Second, information such as charges is transparent, and patients can obtain more information, which is conducive to reducing information asymmetry and improving the relationship between doctors and patients. Third, through a social media-based health management system, patients can actively participate in the system, increasing sustained health engagement [6]. Fourth, after collecting data through a social media-based health management system, analysis can be performed to rationally allocate medical resources and change the state of imbalance of existing medical resources. In short, the benefits of the application of social media-based health management systems are numerous, such as that: (1) They bring convenience to patients’ medical services; (2) they accelerates the transformation of the medical industry; and (3) they make medical services develop in the direction of intelligence, personalization, and autonomy. For instance, Le Zhang et al. proposed the implementation process and significance of developing a medical information service system based on the WeChat public platform. The system is mainly composed of micro-sites and micro-medical networks, which can provide static and dynamic information inquiry services, as well as appointment registration and consulting services. Through this system, patients can receive medical services on the mobile phone in real time, hence simplifying medical procedures and improving patient satisfaction [7]. Further, Haolin studied the performance of social media-based conversation agents in the quit smoking program. The results showed that the presence of social media-based health management systems significantly increased participants’ engagement and smoking cessation effectiveness [8]. What is more, Velasco et al. found that the social media-based mobile Internet health information exchange is regarded as an opportunity to improve public health supervision. On the basis of the traditional systems in which doctors and laboratories report infectious diseases to government agencies, infectious disease cases can be identified more quickly with the help of social media innovation. Social media-based health management systems could allow surveillance epidemiologists to identify potential public health threats, such as rare new diseases and early warning of epidemics [9]. Medical cyber-physical systems (MCPS), which present a new level of integrated intelligence that is characterized by interaction and coordination of computing processes with physical processes, can provide pregnant women with advanced medical care to achieve eugenics [10]. However, one problem of concern is that some patients lose interest in using it and cease using it. Several studies have indicated that users of hospital information systems (HIS) stop using a system after the system has been implemented and adopted by the healthcare organization [11]. Discontinuance wastes a large amount of quality improvement money spent on implementing the system [12]. More importantly, regarding social media-based health management systems (SocialHMS) as a continuous application is important because continuity is the prerequisite for the success of SocialHMS implementations. The significance of behavior continuity in achieving goals has been recognized for a long time in different contexts, including quality improvement [13] and organization success [14]. Thus, it becomes necessary to study factors influencing sustained health engagement and to understand how to use them to enhance the system use and benefits [15].

At present, most of the exiting studies in the area of SocialHMS are focused on the acceptance, development, and application of these systems [16]. In addition, most of the studies about information system (IS) continuance are focused on business-oriented IS such as corporate IS [17,18,19] and e-commerce [20]. Generalizing the outcomes of these studies to the HIS domain is not possible, given the dependence of factors influencing continuance on the context of IS use [21]. Under these circumstances, researchers have called for the study of continuance in the HIS context [22].

Theory of planned behavior (TPB) has been widely accepted as an effective model, which can explain the behavior intention. Further, it is the expansion of theory of reasoned action (TRA), which holds the opinion that any factor could indirectly influence use behavior through attitude and subjective norms [23]. However, the results of many studies show that user’s behavior intention does not always lead to actual behavior. Thus, TPB extends TRA by adding a new component, “perceived behavioral control” to cover nonvolitional behaviors for predicting behavioral intention and actual behavior [24]. According to TPB, individual behaviors can be explained by the behavioral intention which is influenced by attitude, subjective norms, and perceived behavioral control. Attitude towards behavior can reflect likes and dislikes, as well as affective feedbacks, such as whether an experience is pleasant or not [25]. Subjective norm refers to an individual’s perception of social pressure when taking some actions [26]. Perceived behavioral control could be further subdivided into external and internal control factors. Internal control factors refer to an individual’s ability, skill, emotion, the impulse of certain behaviors, etc. External control factors refer to the intervening degree of that environment and facilities for some certain behavior occurring [27]. However, TPB has been criticized for focusing on cognitive factors but ignoring the affect and identity [28].

In the area of social personality psychology, the big five personality types, which are also known as the five-factor model (FFM), have received wide interest and approval [29,30]. This model possesses a high degree of stability in examining cross-cultural phenomena like language benefited from its unique dimensions and levels. Two core issues have been settled in FFM: (1) Distinguishing individual difference and describing order; and (2) structure existing in the individual difference construct [31]. Five relatively stable factors of FFM have been discovered after evaluating and analyzing the experimental results and factors. They are: (1) Extrovert (voluble, confident, and energetic); (2) easy-going (kind, good-natured, cooperative); (3) reliable (cautious, responsible, organized); (4) emotionally stability (calm, non-jittery, good-tempered); and (5) elegant (wise, educated, independent-thinking). All these five factors are highly regarded as the big five, in which the latter has been confirmed by other social-personality psychologists in their strict inspections and studies [32]. From the factors of FFM, openness to experience may be associated with an individual’s behavior intention. The greater the openness of a user, the more willing and confident they are to try new things [33], which reflects an individual’s way of receiving information and processing tendencies.

Perceived risk originates from psychology and has been introduced to the marketing field [34]. It refers to an individual’s perceptions and cognition concerning various objective risks in one’s environment and emphasizes an individual’s experience obtained from intuitive judgment and subjective feeling, as these affect cognitions. Research has found that in certain scenarios, as a result of decision-making variables, behavior intention is related to the level of risk perception of decision makers [35,36].

**Figure 1:**
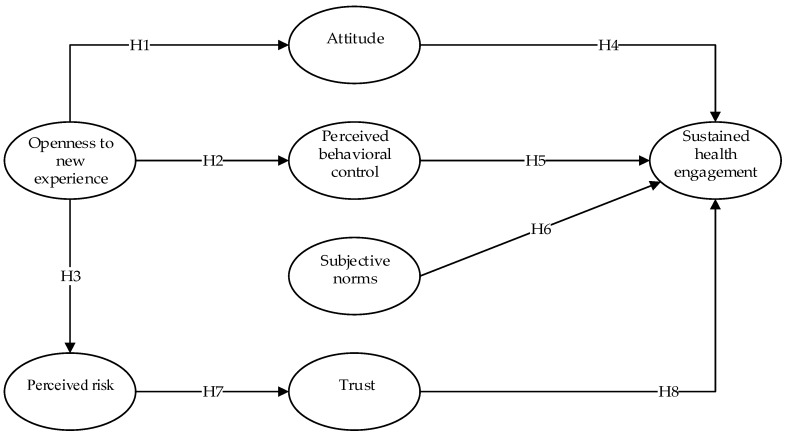
Research model.

To sum up, this study is to investigate the factors influencing continuous use of SocialHMS via the integrated research model which combines TPB with openness experience and perceived risk. The research model is presented in Figure 1. By taking account of numerous literature and actual conditions, we propose eight research hypotheses broken into three categories: (1) Openness to new experience; (2) theory of planned behavior; and (3) perceived risk and trust.

### 1.1. Openness to Experience

Openness to experience, which is from the NEO Personality Inventory (NEO-PI-R) scale defining FFM, is correlated with curiosity, broad-ranging interests, and open-mindedness [37]. Among the factors of FFM, openness to experience may be associated with an individual’s behavior intention because it is a reflection of the characteristics of those individuals who are more open to new ideas and experiences, and more willing and confident to try new things, which reflects an individual’s way of receiving information and processing tendencies. Further, it is a very important variable that can influence the individual’s behavior. Firstly, Lim and Lee found that openness of medical staff and active communication are important for nursing students in order to have a positive attitude toward complementary and alternative therapies [38]. Secondly, Bandura proposed his ternary interaction model that human motivation is generated by one’s behavior, cognitive style, and environment [39]. An individual will not only respond to the external environment, but also adopt some strategies to change the environment they are in. Hascher and Hagenauer examined that openness to experience can positively predict a teacher’s self-efficacy [40]. Hull and Booker’s model also consistently identified openness to experience as a significant contributor to teacher self-efficacy [41]. This research employs the self-efficacy model to evaluate patient performance and feedback on their effectiveness of the SocialHMS. In addition, some research found that the perceived behavioral control based on personal experience and expected blocks will also affect behavior [42]. Thirdly, in the process of using SocialHMS, the user may be confronted with various risks, some of which could be sensed while some could not; some could be exaggerated while some could be diminished. Existing research has shown that perceived risks of consumers from different groups have different impacts on purchase intention [43]. Based on the openness to experience, it is certain that a user with a high degree of openness is good at absorbing new experiences, being brave to take the challenge, and effectively using various strategies to cope with the changes when facing unknowns. Meanwhile, the user of this kind would actively search for innovation when tackling problems, and therefore, can adjust to new changes quickly. In this study, SocialHMS can be viewed as a kind of new experience. These social media-based systems are used in registration, payment, decision-making, chronic diseases management, health information and medical expenses inquiry, etc. A user with a high degree of openness is more willing and confident to accept and use SocialHMS, and they can adjust to the new changes quickly.

Thus, the following are three hypotheses related to openness to experience:

**Hypothesis 1 (H1):**  *Openness to new experience has a significant positive relationship with attitudes towards using the SocialHMS system.*

**Hypothesis 2 (H2):**  *Openness to new experience has a significant positive relationship with perceived behavioral control over use of the SocialHMS system.*

**Hypothesis 3 (H3):**  *Openness to new experience has a significant positive relationship with perceived risk of using the SocialHMS system.*

### 1.2. Theory of Planned Behavior

Perceived behavioral control has been defined as the perceived difficulty of performing a behavior and points out that an individual’s behavior is restricted by his or her own ability to perform the behavior along with influences from the external environment. This reflects one’s cognition about the factors that can either promote or impede the action, thus affecting the possibility to conduct this action. Ajzen cited numerous studies supporting the correlation between the behavior intention and perceived behavior control [42]. He believed that a stronger perceived behavior control led to a stronger behavior intention. Lots of studies showed that perceived behavior control could predict the behavior intention, such as predicting the user’s intention of using the new software [44]. Perceived behavior control could coordinate the relationship between behavior intention and using behavior, thus exerting a direct influence on the behavior intention. Users’ confidence concerning the difficulty of using SocialHMS could influence the capability of controlling the information system, hence affecting their intention to use SocialHMS.

Attitude refers to an individual’s evaluation of personal behaviors. Attitude towards behavior is determined by the product function sum of belief and outcome evaluation. The attitude examined in this research is about the patients’ adaptation to SocialHMS and whether they gained pleasure from it. Healthcare researchers consider attitude as an important factor in determining the user behavior in adopting guidelines or in SocialHMS use. This is evident in influential models created to explain the user’s adoption of guidelines [45] and in attitude being the main predictor and guideline the use in healthcare studies [46,47]. Subjective norm refers to the individual’s perception of what he or she believed about others’ likelihood to sustain use of the SocialHMS [48]. Subjective norms could come from both internal and external avenues. Internal influence mainly refers to an influence that comes from friends, family, colleagues, leadership, etc., while external influence refers to mass media reports, the opinions of the experts but not individual information [49]. Venkatesh and Davis believed that when a social role wants an individual to perform a behavior, and when the social role can provide a corresponding reward or punishment, individuals tend to follow the social impact [50]. Research concerning this influence of social norms comes from examining users who are participating in virtual communities [51]. Bock and Zmud found that organizational climate forms subjective norms and strongly influences knowledge-sharing intentions among workers [52]. Further, the influence of this factor can be used to study the continuous use of IT systems. The use of SocialHMS can be regarded as a kind of social activity, and the users’ behavior toward using SocialHMS can be observed by a range of the group. What is more, behavior toward using SocialHMS may affect the public’s perception and evaluation of users. When both of the two points were perceived by users, it is likely that sustained health engagement will be affected.

Therefore, we proposed the following hypotheses:

**Hypothesis 4 (H4):**  *Attitude has a significant positive relationship with sustained health engagement.*

**Hypothesis 5 (H5):**  *Subjective norms have a significant positive relationship with sustained health engagement.*

**Hypothesis 6 (H6):**  *Perceived behavioral control has a significant positive relationship with sustained health engagement.*

### 1.3. Perceived Risk and Trust

Perceived risk theory believes that as long as the output and outcome are uncertain, risk will be generated [53]. There are two uncertainties of every consequence in the decision to use SocialHMS. One is an uncertainty about the results (whether the results will satisfy their purpose), and the other is uncertainty about the failure of using the system. These uncertainties, when perceived by users, may create situations of risk. For example, some studies have shown that users will give up using the system if they perceive it as a threat to their professionalism [54]. Further, sense of privacy is the aspect of the form of perceived risk on the Internet environment, representing the worries and concerns over one’s exposure of privacy [55]. According to the special use environment of SocialHMS, the patient’s personal privacy information is sometimes recorded. Thus, the protection of privacy information will greatly affect patients’ perceived risk level of SocialHMS. This may then affect patients’ continuous use of the system.

Trust in our study refers to patients’ trust that SocialHMS could provide the service they needed. McKnight had shown that trust has an impact on the intention for continuous use [56]. Based on the earlier research results, trust has the following characteristic, risk. That is to say, trust itself represents the intention to take risks; therefore, our study sought to explore the indirect impact of trust on sustained health engagement through perceived risk.

Thus, we proposed the following hypotheses:

**Hypothesis 7 (H7):**  *Perceived risk has a significant negative relationship with the user trust in SocialHMS.*

**Hypothesis 8 (H8):**  *The user trust in SocialHMS has a significant positive relationship with sustained health engagement.*

## 2. Methodology

### 2.1. Toolkits

The structural equation model (SEM) matured in the 1980s and is a better method in social science research, remedying the shortcomings of traditional statistical methods [57]. Several advantages of SEM are listed as follows: (1) It can process multiple dependent variables at the same time; (2) it allows independent variables and dependent variables to contain measurement errors; (3) it simultaneously estimates factor structure and factor relationship. The measurement of the structural equation model mainly includes two major evaluations. The first is the evaluation of the measurement model, including the relationship between the observed variables and the latent variables (data reliability and validity analysis). The strength of the model’s interpretation of the latent variable is the *R_2_* value. The second is the evaluation of the structural model. That is, the relationship between latent variables supports whether the model hypothesis is supported. The structural equation model is established by partial least squares (PLS), and the measurement quality (measurement model) and mutual relationship (structural model) are evaluated. Though the ordinary least squares method as the estimation technique, PLS performs an iterative factor analysis set and applies a bootstrap method to estimate the significance of the path (*t* value). It is a powerful method for analyzing complex models using smaller samples. SmartPLS is developed in Java and can run on any platform. It provides three choices of internal weight modes: Centroid weight, factor weight, and path weight. It can set the number of iterations, iteration precision, and missing value processing. Thus, in this study, we used SmartPLS to evaluate the measurement properties and tested the hypotheses. Note that SmartPLS is a statistical analysis software designed by the development team of Ring, Wende, and Will of the University of Hamburg, Germany in 2005.

The rationality and reliability of the questionnaire are reflected in the form of the scale. The rationality of the scale determines the reliability and availability of data collection. Therefore, before statistical analysis of the results of the questionnaire, the credibility of the data should be analyzed to ensure the availability of the data and the credibility of the interpretation of the model. Only when the credibility of the data is within the acceptable range are the data collection results of the questionnaire reasonable and reliable, and the value of further analysis and statistics available. Cronbach’s alpha reliability coefficient is used to measure the reliability analysis of the data. The higher the value, the higher the reliability of the table. It is generally accepted that a reliability coefficient above 0.7 is acceptable, and less than 0.7 indicates that the item of the scale needs to be adjusted [58]. Therefore, the threshold used herein is that the Cronbach’s alpha coefficient be greater than 0.7.

Validity analysis, also known as effectiveness analysis, is mainly to detect whether each measurement question accurately expresses the meaning of each research variable [59]. Accurate expression means high degree of agreement, and high degree of data validity. Regarding the analysis of data validity, this study conducted two aspects of analysis, one being the analysis of convergence validity and the other the analysis of differential validity. For convergence validity, this paper measured two aspects: (1) Composite reliability (CR); and (2) average variance extracted (AVE). In general, composite reliability is greater than 0.6, indicating that the inherent consistency of all measurement questions is higher. Average variance extracted (AVE) is greater than 0.5, indicating that the measurement questions can better reflect the characteristics of each research variable in the model [60]. For differential validity, the analysis can be performed by the square root of the AVE value. The data for the diagonal position are the square root of the mean variance extraction rate (AVE value) for each study variable. When the square root of the mean variance extraction rate (AVE value) of each measurement question is greater than the correlation coefficient between the variables [61], it indicates that there is a strong discriminant coefficient between the variables, that is, the difference between each measurement variable is better. In general, the larger the *R^2^* value, the stronger the model’s interpretation of each latent variable.

### 2.2. Sample and Data Collection

Considering the large number of existing studies that have adopted online survey research methods to collect data, we followed a similar data collection method by designing a survey questionnaire to collect data. Measures for the seven variables in our research model were adapted from previous studies. We referred to the relevant research and looked at the variables as well as the relationships among them. Then, by consulting the item of each variable in literature and combining the characteristic of this study, we increased and modified the related question appropriately. Finally, we designed the questionnaire of this study. Our questionnaire adopts a Likert 7-grade scale ranging from 1 (Highly Disagree) to 7 (Highly Agree).

The data collection of this study was carried out in Anhui Medical University first affiliated hospital (AMU), which is a 3A hospital in East China. 3A represents the highest level of hospital in China. The hospital has implemented SocialHMS for 3 years and already has many users. All respondents had 2–3 years of experience using SocialHMS. Firstly, in order to ensure the structural integrity of the questionnaire [62], the English reference scale in the questionnaire was translated into Chinese by a professional translator. After the preliminary design of the questionnaire, in order to ensure the validity and reliability of the questionnaire, we implemented a pilot survey and collected 100 questionnaires in AMU Hospital in June 2014. According to the collected data from the preliminary survey and the advice of the respondents, we adjusted the questions that were difficult to understand. Then, we analyzed the reliability and validity of the data using SmartPLS2.0 and confirmed the good reliability and validity of the questionnaire. The effort to determine reliability is described further along in the data analysis section. Finally, we conducted a survey in the hospital from October 2014 to March 2015. In order to achieve a reasonable response rate, we granted small gifts such as pre-paid phone cards, brush pots, and a creative small fan to each respondent. Each item was valued at about ¥30, about $5. A total of 550 questionnaires were handed out, and we received 532 questionnaires, of which 494 were valid. The return rate was 96.7%, and the valid response rate was 92.9%.

## 3. Results

### 3.1. Analysis of Measurement Model

The acceptability of the measurement model was assessed by the reliability of individual items, internal consistency between items, the model’s convergent, and discriminant validity. Table 1 provides the descriptive statistics generated from the initial data, including the mean, standard deviation, and factor loading for each variable. Table 2 shows the Cronbach’s alpha, composite reliability, average variance extracted (AVE), and square root of the AVE, as well as the correlations between the constructs. It is generally accepted that a reliability coefficient above 0.7 is acceptable. Therefore, the threshold used here is that Cronbach’s alpha coefficient be greater than 0.7 [59]. As shown in Table 2, Cronbach’s alpha of the seven constructs are all above the recommended criterion of 0.70, ranging from 0.7188 (Subjective norm) to 0.8571 (trust), which shows that the measures are reliable and internally consistent. Further, convergent validity and discriminant validity tests can be conducted by using SmartPLS2.0.

Convergent validity is generally assessed by the loadings of all the items; composite reliability (CR), average extracted variance (AVE), and discriminant validity should be evaluated by examining whether AVEs are higher than the interconstruct correlations. As shown in Table 1, the loadings of all the items are above the threshold of 0.75, indicating that the observed variables have high convergent validity. Furthermore, there is a high correlation between the observed variables and their belonging structure variables. Composite reliability that achieved 0.70 or above means the scale has good reliability. In general, composite reliability is greater than 0.6 and average variance extracted (AVE) is greater than 0.5, indicating that the reliability of this model is good [60]. Table 2 shows composite reliability is above 0.70 for all the variables in this study. Moreover, Table 2 shows AVE in this study is above 0.50 throughout, which denotes that the latent variables have a convergence ability that is quite ideal. When the square root of the mean variance extraction rate (AVE value) of each measurement question is greater than the correlation coefficient between the variables, it indicates that the difference between each measurement variable is better [58]. Further, the square root of the latent variables AVE value is greater than the absolute value of the correlation coefficient among latent variables. Thus, the discriminant validity of latent variables has been readjusted to meet standard.

Therefore, the reliability of this study is good.

### 3.2. Analysis of Structural Model

**Figure 2:**
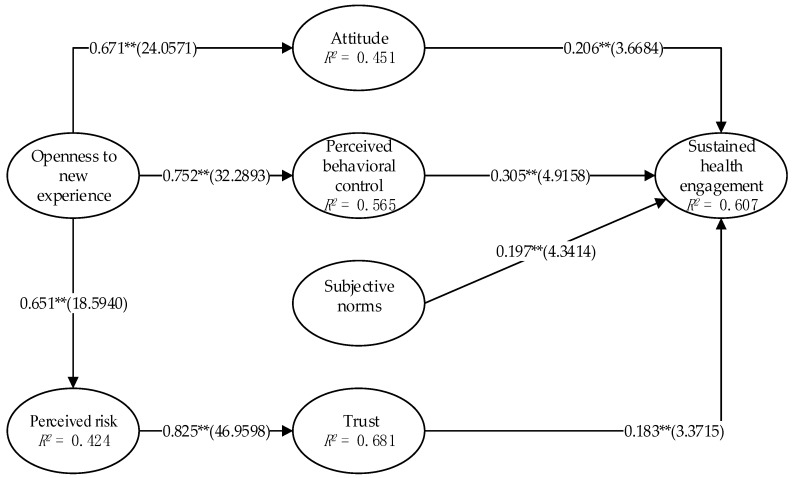
Model results. ** represents *p* < 0.01.

Model fitting results are shown in Figure 2 and Table 3: The hypothesis of the impact of openness to experience personality trait on the attitude of use toward the SocialHMS (H1) is verified. In general, the *t* value is greater than 1.64, indicating that the hypothesis is supported [61]. Its path coefficient was 0.671 (*t* = 24.0571). Obviously, it reaches the significant level of 0.01, and the explained variance is 45.1%. The results show that the independence of the user’s personality has a positive, significant influence on perceived usefulness. The path coefficient of openness to experience to perceived behavioral control is 0.752 (*t* = 32.2893), and this also reaches the significant level of 0.01, with an explained variance of 56.5%. Therefore, openness to experience has a positive significant influence on perceived behavioral control, and (H2) is verified. The path coefficient of openness to experience to perceived risk is 0.651 (*t* = 18.5940), and this also reaches the significant level of 0.01. Because the path coefficient is negative, the negative correlation of openness to experience to perceived risk can be verified. Thus, hypothesis H3 was supported. H7, the hypothesis of perceived risk to trust has been verified, too. Its path coefficient is 0.825 (*t* = 46.9598), reaching the significant level of 0.01, and the explained variance is 68.1%. The path coefficient of attitude (H4), perceived behavioral control (H5), subjective norms (H6), and trust (H8) in the intention of sustained use of the SocialHMS were 0.206 (*t* = 3.6684), 0.305 (*t* = 4.9158), 0.197 (*t* = 4.3414) and 0.183 (*t* = 3.3715), respectively. Therefore, H4, H5, H6, and H8 are verified. H4, H5, H6, and H8 reach the significant level of 0.01. Hence, we drew a conclusion that the perceived risk has a significant influence on the sustained intention to use SocialHMS. The explained variance of the above factors to the sustained health engagement of SocialHMS is 60.7%.

## 4. Discussion

This paper studied the continuous use behavior of SocialHMS. As the experimental analysis results have shown, the 8 hypotheses proposed in the model have all been supported.

The analysis of H1, H2, and H3 showed that the openness to new experience personality trait has a significant positive impact on the attitude, perceived behavioral control, and perceived risk, thus improving sustained health engagement. The verified H4 and H5 show that the attitude and perceived behavioral control have a direct effect on sustained health engagement. The personality trait of openness to experience was a case in point. The greater the openness of a user, the more willing and confident they are to accept and use SocialHMS. Developers can improve SocialHMS based on the openness to experience and match the users through big data and cloud computing with each respective personality characteristic. System designers can improve the operation and function of SocialHMS. Then, system designers can design more humanized and more acceptable SocialHMS to enhance the user’s sustained health engagement. Over time, web designers should seek to improve user attitudes towards, and perceived behavioral control by the use of the SocialHMS, which is likely to increase user engagement.

The empirical results of H6 indicated that subjective norms have a positive effect on the user’s sustained health engagement. By enhancing the user’s subjective norms, through official promotion, advertising, word of mouth, and system promoters can improve patients’ positive cognition of the system [63], as well as effectively enhance the doctors’ sustained health engagement.

The supported H7 indicated that perceived risk has a significant negative relationship with user trust in SocialHMS. The supported H8 shows that user trust in SocialHMS has a significant positive relationship with sustained health engagement. The support for these two hypotheses indicates that the lower the risk in the SocialHMS users perceive, the more trust they place in the system, and the more sustained health engagement will result. From this perspective, hospitals could improve patients’ trust and strengthen their sustained health engagement through improving the SocialHMS safety features and strengthening patients’ privacy safeguards [64].

## 5. Conclusions

Three theories including TPB, the big-five trait theory, and risk and trust theory were introduced in this study so as to, on the one hand, examine the relationship between openness experience and sustained health engagement and, on the other hand, examine how perceived risk affects sustained health engagement in the context of social media-based health management systems (SocialHMS). Our results revealed that attitude, perceived behavioral control, subjective norm, and trust are the determinants that affect sustained health engagement. The users’ personality trait of openness to new experience and perceived risk were also found to be important factors for sustained health engagement. The results of this study integrate two research streams, IS and healthcare, to provide a holistic understanding of the sustained use of SocialHMS both by users and researchers. The research model is quite novel and combines the classic TPB model with both the big-five theory and risk and trust theory. This can be seen as an endeavor to extend the TPB model. In essence, the paper has provided a new perspective to the study of sustained health engagement, demonstrating the effect of personality (openness to new experience) on use of hospital systems [65]. It can also provide designers with a reference for system optimization [66], as well as for decision-making support for medical institutions [67], thus further improving the implementation of various intelligent technologies in hospitals. Designers can improve SocialHMS based on this personality perspective, and they can design more humanized and more acceptable hospital information systems to enhance the user’s sustained health engagement. Through official promotion, advertising, and word of mouth, hospitals can improve patients’ positive cognition of the system and effectively enhance doctors’ sustained health engagement by enhancing the user’s subjective norms. Hospitals could also improve patients’ trust and strengthen their sustained health engagement through improving the SocialHMS safety features and strengthening patients’ privacy safeguards.

## Figures and Tables

**Table 1:** Reliability analysis of variables.

Construct	Item Statistics			
	Construct Items	Mean	Std. Deviation	Loading
Openness to New Experience	OtE1 *	5.44	1.35	0.856783
	OtE2 *	5.64	1.28	0.905741
	OtE3 *	5.26	1.44	0.837521
Attitude	Attitude1	6.07	1.1	0.826909
	Attitude2	6.02	1.1	0.827472
	Attitude3	6.02	1.05	0.848277
	Attitude4	5.93	1.16	0.822302
Perceived Behavioral Control	PBC1 *	5.77	1.2	0.829332
	PBC2 *	5.86	1.19	0.862304
	PBC3 *	5.65	1.28	0.820957
Subjective Norms	SN1	4.96	1.71	0.756089
	SN2	5.62	1.35	0.819427
	SN3	5.55	1.31	0.822804
Perceived Risk	PR1	5.75	1.26	0.891781
	PR2	5.63	1.24	0.917101
Trust	Trust1	5.6	1.23	0.804032
	Trust2	5.44	1.24	0.824332
	Trust3	5.61	1.23	0.847866
	Trust4	5.78	1.19	0.869369
Sustained Health Engagement	SU1	5.69	1.2	0.888886
	SU2	5.62	1.29	0.889958

* The construct items are used to explain the construct. For example, OtE1, OtE2, and OtE3 are scales of the openness to new experience. PBC1, PBC2, and PBC3 are scales of the perceived vehavioral control.

**Table 2:** Validity analysis of variables.

Construct Items	AVE ^1^	Composite Reliability	Cronbach’s Alpha	Attitude	SU	OtE	PBC	PR	SN	Trust
Attitude	0.6911	0.8995	0.851	0.8313 *						
SU	0.792	0.8839	0.7374	0.6929	0.89 *					
OtE	0.752	0.9008	0.835	0.6714	0.655	0.8672 *				
PBC	0.7018	0.8759	0.788	0.7796	0.7148	0.7515	0.8377 *			
PR	0.8182	0.9	0.7786	0.6281	0.6234	0.651	0.654	0.9045 *		
SN	0.64	0.8419	0.7188	0.6269	0.6351	0.6408	0.6208	0.6024	0.8 *	
Trust	0.7002	0.9032	0.8571	0.6897	0.6661	0.713	0.6961	0.8255	0.6562	0.8368 *

^1^ AVE stands for average variance extract. * The bold number is the square root of AVE. The bold numbers listed diagonally are the square root of the variance shared between the constructs and their measures. The off-diagonal elements are the correlations among the constructs. For discriminate validity, the diagonal elements should be larger than the off-diagonal elements.

**Table 3:** Hypothesis testing results of the model.

Hypothesized Path.	Standardized Path Coefficients	*t*-Value	Results
H1: OtE -> Attitude	0.671	24.0571	Yes
H2: OtE -> PBC	0.752	32.2893	Yes
H3: OtE -> PR	0.651	18.594	Yes
H4: Attitude -> SU	0.206	3.6684	Yes
H5: PBC -> SU	0.305	4.9158	Yes
H6: SN -> SU	0.197	4.3414	Yes
H7: PR -> Trust	0.825	46.9598	Yes
H8: Trust -> SU	0.183	3.3715	Yes
